# A Medical Vocabulary

**Published:** 1837-01

**Authors:** 


ART. X.—*A Medical Vocabulary; or, Explanation of all Names, Synonymes, Terms and Phrases used in Medicine, Surgery, and the relative Branches of Medical Science; giving the correct Derivation and Directions for the proper Pronunciation of each; intended chiefly as a Book of Reference for the Student, but also adapted to general Use.* BY A MEDICAL PRACTITIONER.—*Edinburgh,* 1836. 12mo. pp. 185.

THE small book with the long title which we have just transcribed, is, without any exception, the best of its kind which we have yet seen. It seems to be the very thing which was wanting for the student; and it will, moreover, be found useful to every medical reader, as no one can carry in his head the whole *technicology* of medicine. We have carefully looked through the pages of this little work, and must say that it is, in our opinion, well conceived and cleverly and carefully executed. We know not who the author is, but he has certainly laid the whole class of medical students under obligations to him. The great advantage which it has over all other works of the kind which we have seen, is in the perfect pertinency and conciseness of the explanations given of the terms. Nothing superfluous is admitted, and we find as few deficiencies as errors, though we have noted a few of both. The derivations of the words are also, on the whole, very correct. If the synonymes were given in all the modern languages (and they are rarely given in any) this vocabulary would go some way towards realizing (as far as England is concerned) the projected work announced in our last number (p. 510) in our review of Dr. Palmer's dictionary. The following is the plan of the vocabulary, as given in the preface: "the name, term, or phrase is set down with marks for its proper pronunciation—the long, short, and acute accents; if a Latin word, simple or compound, its declension is given; next its derivation, and the reason therefor, where the connexion may not be very apparent; then its character, and general and particular meaning, with the corresponding word or term in Latin or English; lastly, its synonyms."

